# Early Activation of STAT3 Regulates Reactive Astrogliosis Induced by Diverse Forms of Neurotoxicity

**DOI:** 10.1371/journal.pone.0102003

**Published:** 2014-07-15

**Authors:** James P. O'Callaghan, Kimberly A. Kelly, Reyna L. VanGilder, Michael V. Sofroniew, Diane B. Miller

**Affiliations:** 1 Centers for Disease Control and Prevention, National Institute for Occupational Safety and Health, Morgantown, West Virginia, United States of America; 2 School of Pharmacy, West Virginia University, Morgantown, West Virginia, United States of America; 3 David Geffen School of Medicine, University of California Los Angeles, Los Angeles, California, United States of America; University of Nebraska Medical Center, United States of America

## Abstract

Astrogliosis, a cellular response characterized by astrocytic hypertrophy and accumulation of GFAP, is a hallmark of all types of central nervous system (CNS) injuries. Potential signaling mechanisms driving the conversion of astrocytes into “reactive” phenotypes differ with respect to the injury models employed and can be complicated by factors such as disruption of the blood-brain barrier (BBB). As denervation tools, neurotoxicants have the advantage of selective targeting of brain regions and cell types, often with sparing of the BBB. Previously, we found that neuroinflammation and activation of the JAK2-STAT3 pathway in astrocytes precedes up regulation of GFAP in the MPTP mouse model of dopaminergic neurotoxicity. Here we show that multiple mechanistically distinct mouse models of neurotoxicity (MPTP, AMP, METH, MDA, MDMA, KA, TMT) engender the same neuroinflammatory and STAT3 activation responses in specific regions of the brain targeted by each neurotoxicant. The STAT3 effects seen for TMT in the mouse could be generalized to the rat, demonstrating cross-species validity for STAT3 activation. Pharmacological antagonists of the neurotoxic effects blocked neuroinflammatory responses, pSTAT3^tyr705^ and GFAP induction, indicating that damage to neuronal targets instigated astrogliosis. Selective deletion of STAT3 from astrocytes in STAT3 conditional knockout mice markedly attenuated MPTP-induced astrogliosis. Monitoring STAT3 translocation in GFAP-positive cells indicated that effects of MPTP, METH and KA on pSTAT3^tyr705^ were localized to astrocytes. These findings strongly implicate the STAT3 pathway in astrocytes as a broadly triggered signaling pathway for astrogliosis. We also observed, however, that the acute neuroinflammatory response to the known inflammogen, LPS, can activate STAT3 in CNS tissue without inducing classical signs of astrogliosis. Thus, acute phase neuroinflammatory responses and neurotoxicity-induced astrogliosis both signal through STAT3 but appear to do so through different modules, perhaps localized to different cell types.

## Introduction

Astrogliosis, the “reactive” state of astrocytes, is a pathological hallmark of all types of CNS injuries [1]–[5]. A dominant feature of astrogliosis is cellular hypertrophy with an attendant accumulation of GFAP-enriched intermediate filaments [3], . Different types of injuries and multiple molecular signaling pathways are able to trigger this common feature of astrocytic reactivity. Nevertheless, a wide spectrum of potential molecular and cellular features of astrogliosis also have been described [8], [9]; qualitatively dissimilar insults of varying severity (e.g., neurodegenerative disease, neurotrauma or neurotoxicity) may engender varying degrees of astrogliosis with different morphological features, molecular underpinnings and functional consequences [8]–[10]. Thus, focusing on limited numbers of injury models to investigate astrogliosis may lead to generalizations that do not apply to all types of astroglial responses to injury.

Here we examined signaling events associated with astrocytic responses to neural damage resulting from diverse neurotoxic insults. Using neurotoxicants to instigate astrogliosis is advantageous because different brain regions and often, different cell types within a given region, can be selectively targeted. Previously, we found that different neurotoxicants result in brain-region-specific astrogliosis tightly linked to the damaged target, as well as the dose, time and duration of the neurotoxic effect, regardless of the region or cell type affected [11]. Because systemic administration of these neurotoxicants often does not damage the blood-brain barrier (BBB), the observed astroglial responses are not complicated by contribution of blood-borne factors at the sites of damage, as is the case for other models. Moreover, pharmacological and genetic manipulations of neurotoxicity reveal that neurotoxic injuries of neuronal targets serve as the stimulus to initiate astrogliosis, thereby ruling out direct effects on the astrocytes themselves. Using diverse neurotoxicity models to delineate common signaling mechanisms responsible for instigating astrogliosis offers an approach for pursuit of therapeutic interventions based on manipulating astroglial reactivity.

Previously, we showed that MPTP-induced dopaminergic neurotoxicity was linked to rapid but transient phosphorylation of STAT3^Tyr 705^, and its translocation to astrocytic nuclei, prior to the induction of GFAP mRNA and protein [12]. Enhanced expression of proinflammatory signaling through the JAK2-STAT3 pathway was observed prior to onset of STAT3 activation. Other proinflammatory mediators known to feed into the JAK-STAT3 signaling cascade (e.g., TNF-α and IL-1β) [13] also showed enhanced expression prior to phosphorylation of STAT3 and induction of GFAP [14]. These findings suggested that neuroinflammation-mediated activation of the STAT3 pathway may be associated with induction of astrogliosis, findings consistent with prior observations for a role of STAT3 in astroglial reactivity and scar formation resulting from neurotrauma and ischemia [15]–[19]. Here we sought to determine the role of enhanced astrocytic STAT3 signaling in multiple mechanistically distinct models of neurotoxicity. Because proinflammatory signaling in the CNS can occur in the absence of neural damage [20]–[23] we also examined whether such neuroinflammatory responses could activate STAT3 without inducing astrogliosis. While our findings strongly implicate astroglial STAT3 activation as a common feature in all of our neurotoxicity models, we also observed that acute neuroinflammatory responses to the known inflammogen, LPS, can activate STAT3 without inducing GFAP up-regulation, a hallmark of astrogliosis. Thus, acute phase neuroinflammatory responses and neurotoxicity-induced astrogliosis signal through STAT3 but appear to do so through different STAT3 modules.

## Materials and Methods

The following drugs and chemicals were kindly provided by or obtained from the sources indicated: lipopolysaccharide (LPS; Sigma Chemical Co., St. Louis, MO), 1-methyl-4-phenyl-1,2,3,6-tetrahydropyridine (MPTP; Aldrich, Milwaukee, WI), methamphetamine (METH; Sigma), amphetamine (AMP; Sigma), 3,4-methylenedioxymethamphetamine (MDMA; Research Technology Branch of the National Institute on Drug Abuse, Rockville, MD), 3,4-methylenedioxyamphetmine (MDA; Research Technology Branch of the National Institute on Drug Abuse), kainic acid (KA; Sigma), trimethyltin (TMT; K&K Laboratories, Division of ICN Biochemical, Cleveland, OH), nomifensine (Sigma), Ethanol (Sigma), diazepam (Sigma), minocycline (Sigma), corticosterone (CORT; Steraloids, Inc., Newport, RI), bicinichoninic acid protein assay reagent and bovine serum albumin (Pierce Chemical Co., Rockford, IL). The materials used in the glial fibrillary acidic protein (GFAP) ELISA have been described in detail [24], [25]. The materials used in the tyrosine hydroxylase (TH) ELISA have been described previously [12], [26], [27]. All other reagents and materials were of at least analytical grade and were obtained from a variety of commercial sources.

### Animals

Studies on MPTP, METH, AMP, MDMA, MDA and TMT used male C57BL/6J mice; studies on KA used male FVB/NJ mice; LPS studies used female C57BL/6J mice. An additional study on TMT used male Long-Evans rats where indicated. C57BL/6J or FVB/NJ mice (n = 5 mice per group) 4–6 weeks of age were purchased from Jackson Labs (Bar Harbor, ME). Long-Evans male rats were purchased from Charles River Laboratories International, Inc. (Wilmington, MA).

Mice selectively deficient in STAT3 in astrocytes (STAT3-CKO) using GFAP promoter-directed Cre/loxP technology as described previously [17], [28] were used for GFAP and TH protein quantification after MPTP exposure.

All procedures were performed within protocols approved by the Institutional Animal Care and Use Committee of the Centers for Disease Control and Prevention, National Institute for Occupational Safety and Health, and the animal colony was certified by the American Association for Accreditation of Laboratory Animal Care. Upon receipt, male mice were housed individually and females were housed in groups of 6 in a temperature-controlled (21±1°C unless otherwise stated) and humidity-controlled (50±10%) colony room maintained under filtered positive-pressure ventilation on a 12 h light/12 h dark cycle beginning at 0600 EDT. The plastic tub cages were 46 cm in length by 25 cm in width by 15 cm in height; cage bedding consisted of heat-treated pine shavings spread at a depth of approximately 4 cm. Food (Purina rat/mouse chow) and water were available *ad libitum*.

### Dosing

Neurotoxicants were administered to mice as follows: MPTP (12.5 mg/kg, s.c.), AMP (10 mg/kg, s.c., 3 injections at 2 h intervals), METH (20 mg/kg, s.c., 3 injections at 2 h intervals (specified as multi-dose in appropriate figure legends) or a single dose of 20 mg/kg, s.c. (specified as single dose in appropriate figure legends), MDA (10 mg/kg, s.c., 3 injections at 2 h intervals), MDMA (20 mg/kg, s.c., 3 injections at 2 h intervals), KA (20 mg/kg, s.c.) and TMT (0.5 mg/kg, i.p.). TMT was administered to rats at a dosage of 8.0 mg/kg, i.p. All dosages were administered as the base compound.

Pharmacologic and environmental manipulations were administered to mice as follows: Nomifensine (25 mg/kg, s.c.) was given 30 min before MPTP, Ethanol (3 g/kg, s.c.) was given 30 min before METH (20 mg/kg, s.c.), Diazepam (30 mg/kg, i.p.) was given 30 min before KA, and CORT (20 mg/kg, s.c.) was given 30 min prior to MPTP, METH, TMT or LPS. Mice were kept at 15°C for 24 hours and then treated with METH (20 mg/kg, s.c.) and kept at 15°C until killed.

LPS was used as a known inflammogen and administered at a dosage of 2 mg/kg, s.c. or, in the dose-response experiment, at a dosage of 1–5 mg/kg, i.p. [5].

The post-dosing time points were chosen to capture peak expression of cytokines (6–12 hrs.), pSTAT3 (12 hrs) and GFAP (12–72 hrs) based on our previous findings for MPTP [12], substituted amphetamines [29], LPS (unpublished data) and our historical data for induction of GFAP on all compounds used (e.g., see [30]).

### Brain dissection and tissue preparation

Mice were killed by decapitation and whole brains were removed from the skull with the aid of blunt curved forceps. Striatum, hippocampus, cortex, cerebellum and olfactory bulb were dissected free hand on a thermoelectric cold plate (Model TCP-2, Aldrich Chemical Co., Milwaukee, WI) using a pair of fine curved forceps (Roboz, Washington, DC). Brain regions from one side of the brain were frozen at -85°C and used for subsequent isolation of total RNA; brain regions from the other side of the brain were used for total and specific protein analysis. The right-side brain regions were weighed, homogenized with a sonic probe (model XL-2005, Heat Systems, Farmingdale, NY) in 10 volumes of hot (90–95°C) 1% sodium dodecyl sulfate, and stored frozen at −70°C before total protein assay and immunoassay of GFAP and TH.

A separate set of mice were used for pSTAT3^tyr 705^ quantification and were killed by focused microwave irradiation (Muromachi Kikai, Inc., Tokyo, Japan; Model TMW-4012C, 3.5 KW applied power, 0.90 sec) to preserve steady-state protein phosphorylation [31]. Brains were dissected freehand, weighed and homogenized in 10 volumes of hot 1% SDS and then stored at −80°C until assayed. We note that while this mode of sacrifice may be essential for preservation of phosphorylation of many phosphoproteins [31], [32], pSTAT3^tyr 705^ can successfully be preserved by rapid decapitation and sample denaturation in hot 1% SDS [31].

For immunohistochemistry experiments, animals were transcardially perfused with saline (0.9%) followed by formalin (10%) to fix the brain tissue. Brains were removed and kept in formalin (10%) until embedded in paraffin. Tissue was cut and placed in Paraform cassettes (Sakura Finetek, Torrance, CA) for subsequent processing by dehydration, clearing and infiltration overnight with paraffin using a Tissue-Tek VIP 5 Vacuum Infiltration Processor (Sakura Finetek). Briefly, the brains were processed in 10% formalin, 70%, 80%, 95% and 100% ethanol, xylene and then paraffin. The cassette with paraffin-embedded tissue was then attached to a mold (Sakura Finetek) with paraffin.

### RNA isolation, cDNA synthesis, and real-time PCR amplification

Total RNA from striatum, hippocampus, cortex, cerebellum and hypothalamus were isolated using Trizol reagent (Invitrogen, Grand Island, NY) and Phase-lock heavy gel (Eppendorf AG, Hamburg, Germany). The RNA was further cleaned using RNeasy mini spin column (Qiagen, Valencia, CA). Total RNA (1 µg) was reverse transcribed to cDNA using SuperScript II RNase H^−^ and oligo (dT)_12-18_ primers (Invitrogen) in a 20 µL reaction. Real-time PCR analysis of Glyceraldehyde-3-phosphate dehydrogenase (*Gapdh*), tumor necrosis factor-alpha (*Tnf-α*), chemokine (C-C motif) ligand 2 (*Ccl2*), Leukemia inhibitor factor (*Lif*), Oncostatin M (*Osm*) and glial fibrillary acidic protein (*Gfap*) was performed in an ABI PRISM 770 sequence detection system (Applied Biosystems, Carlsbad, CA) in combination with TaqMan chemistry. Specific primers and dual-labeled internal fluorogenic (FAM/TAMRA) probe sets (TaqMan Gene Expression Assays) for these genes were procured from Applied Biosystems and used according to the manufacturer's recommendations. All PCR amplifications (40 cycles) were performed in a total volume of 50 µL, containing 1 µL cDNA, 2.5 µL of the specific Assay of Demand primer/probe mix, and 25 µL of Taqman Universal master mix. Sequence detection software (version 1.7; Applied Biosystems) results were exported as tab-delimited text files and imported into Microsoft Excel for further analysis. Relative quantification of gene expression was performed using the comparative threshold (C_T_) method as described by the manufacturer (Applied Biosystems; User Bulletin 2). Changes in mRNA expression levels were calculated after normalization to *Gapdh*. The ratios obtained after normalization are expressed as fold change over corresponding saline-treated controls.

### Protein assay

Total protein was determined by the bicinchoninic acid method [33] using bovine serum albumin as standard.

### GFAP and TH assays

GFAP was assayed in accordance with a previously described ELISA [24], [25]. In brief, a rabbit polyclonal antibody to GFAP (1∶300; DAKO, Carpenteria, CA) was coated on the wells of Immulon-2 microliter plates (Thermo Labsystems, Franklin, MA). The sodium dodecyl sulfate homogenates and standards were diluted in phosphate-buffered saline (pH 7.4) containing 0.5% Triton X-100. After blocking non-specific binding with 5% non-fat dairy milk, aliquots of the homogenate and standards were added to the wells and incubated. Following washes, a mouse monoclonal antibody to GFAP (1∶200; EMD Millipore-Calbiochem, Billerica, MA) was added to ‘sandwich’ the GFAP between the two antibodies. An alkaline phosphatase-conjugated antibody directed against mouse IgG (1∶2000; Jackson ImmunoResearch, West Grove, PA) was then added and a colored reaction product was obtained by subsequent addition of the enzyme substrate p-nitrophenol (Bio-Rad Laboratories, Hercules, CA). Quantification was achieved by spectrophotometry of the colored reaction product at 405 nm in a microplate reader, Spectra Max Plus, and analyzed using Soft Max Pro Plus software (Molecular Devices, Sunnyvale, CA). The amount of GFAP in the samples was calculated as micrograms of GFAP per milligram total protein.

TH holoenzyme protein was assessed using a fluorescence-based ELISA developed in the laboratory [12]. The protocol was essentially similar to that for the GFAP assay except that a mouse monoclonal antibody to TH (1∶400; Sigma-Aldrich, St. Louis) was used as the plate capture antibody and a rabbit polyclonal antibody (EMD Millipore-Calbiochem, Billerica, MA) was used to ‘sandwich’ TH protein. The amount of sandwich antibody bound to TH was then detected using a peroxidase-labeled antibody directed against rabbit IgG (Artisan Technology Group, Champaigne, IL). Peroxidase activity was detected using the fluorogenic substrate Quantablu (Pierce), which has excitation and emission maxima of 325 and 420 nm, respectively (read at 320/405 nm). The amount of TH in the samples was calculated and expressed as micrograms TH per milligram total protein.

### pSTAT3 immunoblot analysis

Activation of the STAT3 pathway was assessed by quantifying pSTAT3^tyr705^ using immunoblot analysis with detection of fluorescent signals using an infrared fluorescence scanner (Licor Biosciences, Lincoln, NE) or with detection using an ECL chemiluminescent substrate (Amersham Biosciences, Piscataway, NJ) captured onto x-ray film (Fuji Medical systems, Stamford, CT) as described previously [12], [19]. Briefly, following incubation with primary antibodies (anti-phospho-STAT3^tyr705^[1∶500]), blots were washed with phosphate buffered saline with 0.1% Tween 20 and incubated with fluorescent-labeled secondary antibodies (1∶2500) for 1 h prior to scanning by Licor or using a Personal Densitometer (Molecular Dynamics, Sunnyvale, CA).

### Histology

For immunohistochemical analysis of STAT3 and GFAP the paraffin embedded brains were sectioned with a manual rotary Microm microtome (Thermo Scientific, Kalamazoo, MI) into 6 µm sections and floated onto Superfrost + slides (Fisher Scientific). The slides were warmed at 60°C for 20 min. To dissolve away the paraffin, slides were put through xylene and then 100% followed by 95% ethanol washes. Following an overnight incubation in primary antibody STAT3 (rabbit;1∶300; Cell Signaling Technology, Inc., Danvers, MA); GFAP (chicken;1∶300; Abcam, Cambridge, MA) sections were rinsed in PBS and incubated with secondary antibody (for STAT3, 1∶250; for GFAP 1∶250) for 4 hours at room temperature. The sections were rinsed and coverslipped with prolong gold antifade with DAPI mountant (Life Technologies, Grand Island, NY). The sections were visualized using an Olympus AX70 microscope with a PlanApo 40x 0,85 NA objective lens and images captured using Cell Sens Dimension software with the Olympus DP73 digital camera attached to the microscope. Post-processing of images was done according to accepted practices and image integrity guidelines (e.g., [34], [35]). Specifically, the tone was normalized in all images and STAT3 images were sharpened.

### Statistics

All analyses were performed using SigmaPlot (version 11) statistical software. The test of significance was performed using either one or two way ANOVA followed by Fisher LSD test of log transformed data. Values were considered statistically significant at 5% level of significance (p<0.05). Graphical representations are mean ± SEM.

## Results

### STAT3 activation coincides with the onset of damage and precedes GFAP up regulation in multiple models of striatal neurotoxicity

**Figure 1:**
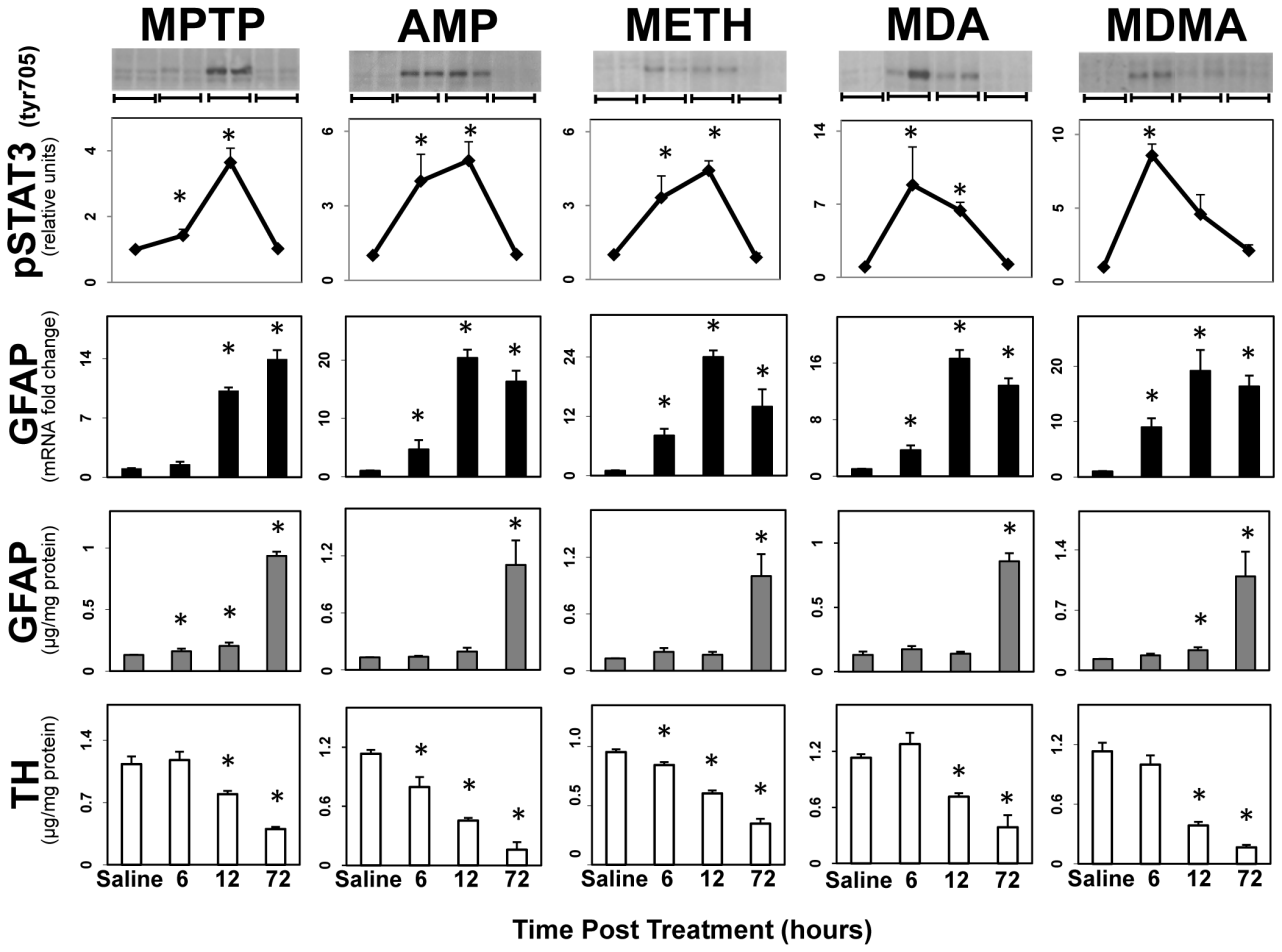
STAT3 activation precedes GFAP up regulation in multiple models of striatal neurotoxicity. Mice were administered dopaminergic neurotoxicants, MPTP, AMP, METH, MDA and MDMA with saline (0.9%) as a control and were killed at 6, 12 and 72 hours post dosing, time points known to encompass the onset of dopaminergic neurotoxicity and the subsequent activation of microglia and astroglia [12]. Mice were killed by focused microwave irradiation to preserve steady-state phosphorylation of pSTAT3^tyr 705^ and phosphorylation was analyzed by quantification of scans of pSTAT3^tyr705^ immunoblots. Representative immunoblot data from two different animals killed at each time point are presented above the quantitative data obtained from scans and are denoted by a bracket. Separate groups of mice were used to prepare total RNA from one side of the brain for qRT-PCR analysis of *Gfap* mRNA and to prepare total protein homogenates from the other side of the brain for ELISA of GFAP and TH. Except for the representative immunoblot duplicates, all data points represent mean ± SEM, N = 5. Statistical significance was measured by one-way ANOVA with Fisher's LSD Method of *post hoc* analysis. Statistical significance of at least p<0.05 for the neurotoxicant exposed groups in comparison to saline controls is denoted by *. See Methods for dosing regimen (multi-dose METH).

Using the known dopaminergic neurotoxicant, MPTP, as a chemical denervation tool, we previously demonstrated that activation of the JAK2/STAT3 pathway in astrocytes, *in vivo*, precedes the up regulation of the astrocyte intermediate filament protein, GFAP, a hallmark of astrogliosis [12]. If the JAK2/STAT3 pathway is a key to induction of astrogliosis, then like astrogliosis, phosphorylation of STAT3^tyr705^ should be associated with multiple models of CNS neurotoxicity. To assess this possibility we began by examining the time-course of STAT3 phosphorylation in relation to the expression of GFAP following the administration of multiple neurotoxic insults known to damage dopaminergic nerve terminals in the striatum (Fig. 1). In agreement with our prior findings, MPTP resulted in a time-dependent decrease in striatal TH consistent with the loss of dopaminergic terminals. Coincident with this nerve terminal damage, STAT3 was activated by phosphorylation on Tyr 705, followed by the induction of *Gfap* mRNA, and the enhanced expression of GFAP. Amphetamine and a series of its substitutes (METH, MDA, and MDMA), all also known to damage dopaminergic terminals in the mouse [29], [36], showed the same relationship among loss of TH, activation of STAT3, and induction of GFAP (mRNA and protein). Dexfenfluramine, an amphetamine that does not damage the striatum [29], [36], did not activate STAT3 or induce the expression of GFAP (data not shown). Non-phospho STAT3 levels were not affected by these dopaminergic neurotoxicants.

### STAT3 activation precedes GFAP up regulation in diverse models of damage affecting multiple brain areas

**Figure 2:**
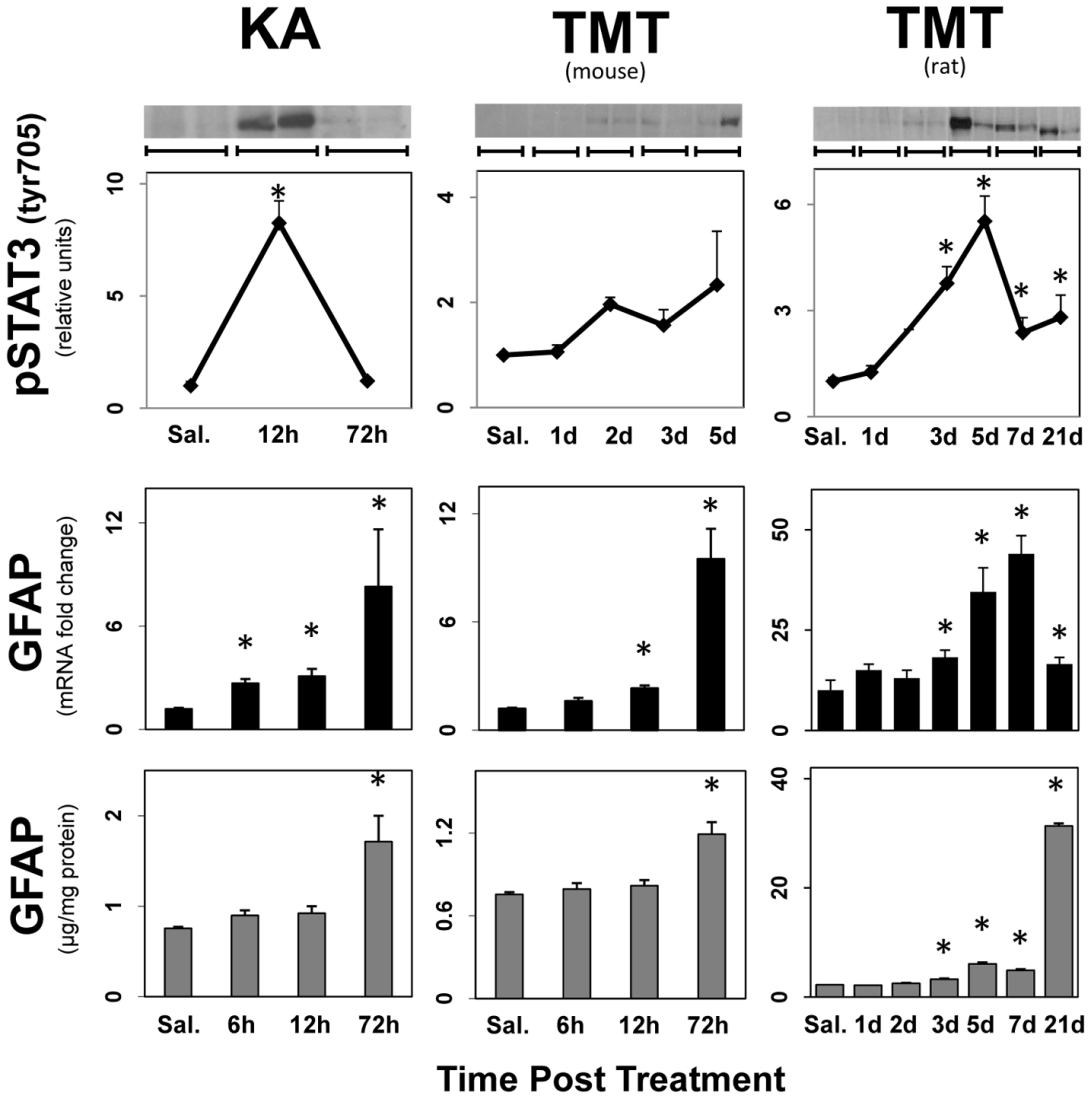
STAT3 Activation precedes GFAP up regulation in diverse models of damage affecting different brain areas. Mice were administered the hippocampal neurotoxicant KA and mice and rats were administered the hippocampal neurotoxicant, TMT. Mice were killed by focused microwave irradiation to preserve steady-state phosphorylation of pSTAT3^tyr 705^ and phosphorylation was analyzed by quantification of scans of pSTAT3^tyr705^ immunoblots. Representative immunoblot data from two different animals killed at each time point are presented above the quantitative data obtained from scans and are denoted by a bracket. Separate groups of mice were used to prepare total RNA from one side of the brain for qRT-PCR analysis of *Gfap* mRNA and to prepare total protein homogenates from the other side of the brain for ELISA of GFAP. Except for the representative immunoblot duplicates, all data points represent mean ± SEM, N = 5 (with the exception of pSTAT3 expression in TMT treated mice in which N = 2). Statistical significance was measured by one-way ANOVA with Fisher's LSD Method of post hoc analysis. Statistical significance of at least p<0.05 for the neurotoxicant exposed groups in comparison to saline controls is denoted by *. See Methods for dosing regimen.

If activation of STAT3 represents a signaling mechanism common to astrogliosis, it should occur in any of the brain regions damaged by the varied types of neurotoxic insults. To address this issue we administered neurotoxicants known to damage the hippocampus and cause an ensuing astrogliosis (Fig. 2). We found that the excitotoxic compound, KA, as well as the organotin neurotoxicant, TMT, resulted in an enhanced expression of GFAP preceded by activation of STAT3 in hippocampus. TMT causes hippocampal damage in both mice and rats and we observed enhanced expression of GFAP preceded by activation of STAT3 in the rat, and a trend toward activation of STAT3 in the mouse that did not reach significance, likely due to a low N. Collectively, these data show that phosphorylation of STAT3^tyr 705^ is associated with the induction of astrogliosis in multiple models of neurotoxicity affecting different brain regions and causing damage via different mechanisms. As with the dopaminergic neurotoxicants, the increases in phospho-STAT3^tyr 705^ could not be attributed to increases in STAT3.

### STAT3 activation is localized to astrocytes after striatal and hippocampal neurotoxicity

**Figure 3:**
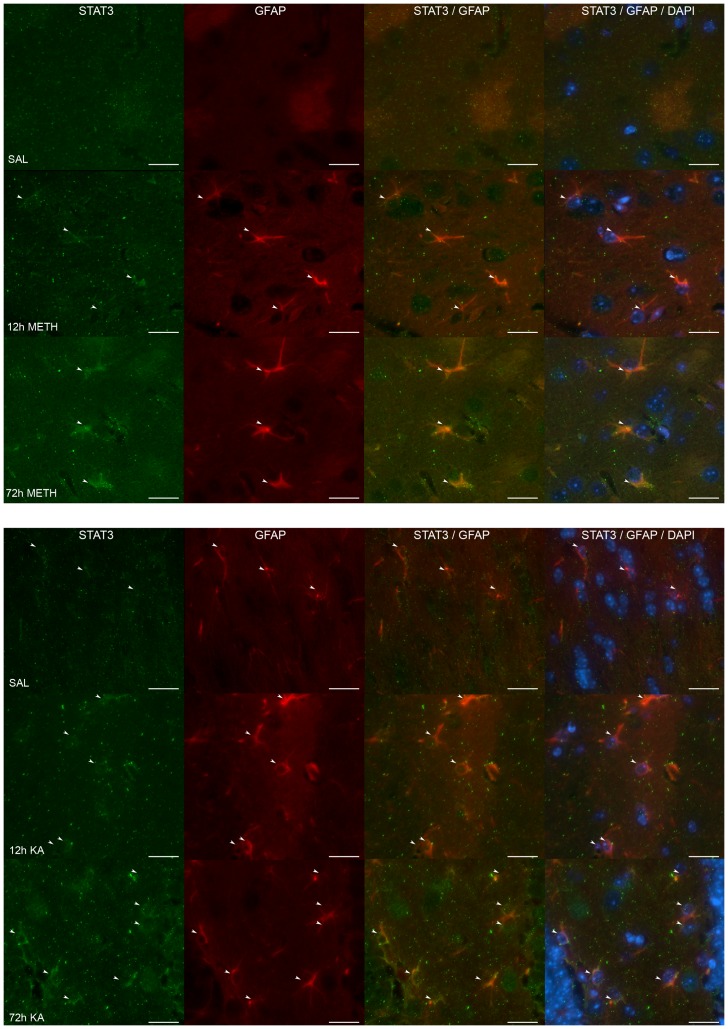
STAT3 Activation is localized to astrocytes after hippocampal as well as striatal neurotoxicity. Mice were administered METH or KA and were processed for immunohistochemical analyses of GFAP to identify astrocytes (denoted by arrows) and STAT3. GFAP immunostaining revealed hypertrophied striatal astrocytes at 12 hours after METH and were further evident by 72 hours. STAT3 staining of striatal nuclei indicative of its translocation and, therefore, activation, was apparent at 72 hours; STAT3 activation was localized to astrocytes as evidenced by an increase in nuclear stating in the merged images at 12 and 72 hours. STAT3 and GFAP staining were not evident in saline-treated striatum sections. In the hippocampus, GFAP immunostaining revealed astrocytes in saline-treated mice; enhanced immunostaining of astrocytes was evident by 72 hours after administration of KA. STAT3 staining of nuclei in hippocampus was prominent at 12 and 72 hours after KA but not evident in saline controls; merging of GFAP and STAT3 images showed increased nuclear staining of GFAP positive cells at 12 and 72 hours post KA suggestive of the translocation of and activation of STAT3 in astrocytes. Merge of GFAP and STAT3 is shown with DAPI for clarity of nucleus location. Arrows corresponding to GFAP positive astrocytes are included in each panel to focus on the points of interest. Scale bars  = 20 µm. See Methods for dosing regimens.

Previously, we showed that damage to dopaminergic nerve terminals from a single dose of the dopaminergic neurotoxicant, MPTP, resulted in striatal-selective increases in GFAP and activation of STAT3 localized to astrocytes. Here we determined if astrocytic localization of STAT3 also would be associated with the astrocytic response to METH-induced dopaminergic neurotoxicity in striatum and KA-induced neurotoxicity in hippocampus (Fig. 3). Damage to dopaminergic nerve terminals in striatum by a single 20 mg/kg dose of METH resulted in enhanced immunostaining of GFAP (denoted by arrows), consistent with astrogliosis and the previously documented increases in striatal GFAP [27], [29], [37]. Enhanced GFAP staining was detected as early as 12 hours after METH. STAT3 immunostaining was used to gauge translocation and enrichment of nuclear STAT3 in astrocytes [12]. Merging of GFAP and STAT3 and GFAP and STAT3 with DAPI immunostaining showed enhanced nuclear and perinuclear staining at both 12 and 72 hours after METH. STAT3 staining alone showed localization to the nucleus by 72 hours after METH. As with the damage to striatum engendered by MPTP and amphetamines, damage to hippocampus by a single 20 mg/kg dose of KA resulted in enhanced immunostaining of GFAP (denoted by arrows; Fig. 3) consistent with the previously documented increase in hippocampal GFAP (Fig. 2). Enhanced GFAP immunostaining was observed as early as 12 hours after KA and increased by 72 hours after KA. Merging of GFAP and STAT3 and GFAP and STAT3 with DAPI immunostaining showed enhanced nuclear staining localized to astrocytes that appeared to increase between 12 and 72 hours after KA. STAT3 staining alone was largely nuclear and was most apparent at 72 hours after administration of KA. STAT3 immunostaining was not observed in saline controls for striatum or hippocampus. Together, these observations are consistent with an astrocytic localization of activated STAT3 in association with the induction of astrogliosis.

### STAT3 activation does not occur in non-damaged brain regions in multiple models of neurotoxicity

**Figure 4:**
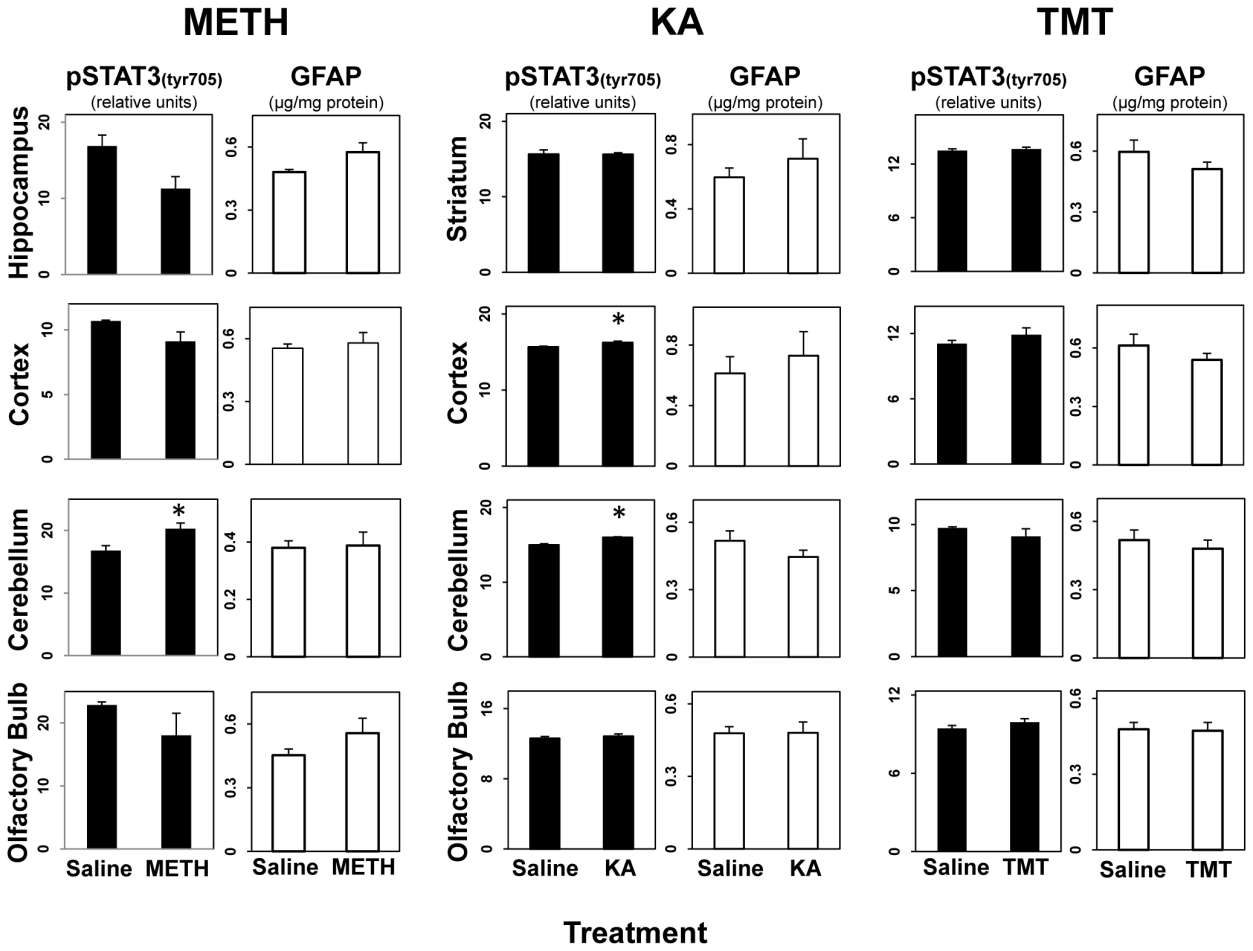
STAT3 Activation does not occur in non-damaged brain regions in multiple models of neurotoxicity. Mice were administered METH, KA or TMT with saline (0.9%) as a control and were killed by focused microwave irradiation at 12 hrs. post dosing and by decapitation at 72 hours post dosing. Multiple “non-target” brain regions for each neurotoxicant were sampled for activated STAT3 (12 hours post dosing) and levels of GFAP (72 hours post dosing). All data points represent mean ± SEM, N = 5. Statistical significance was measured by one-way ANOVA. Where significant differences were found, Fisher's LSD Method of post hoc analysis was performed. Statistical significance of at least p<0.05 for the neurotoxicant exposed groups in comparison to saline controls is denoted by *. See Methods for dosing regimen (multi-dose METH).

Toxicant-induced astrogliosis is restricted to the onset, duration, and location of damage [11]. Therefore, areas of the CNS not affected by a given neurotoxic exposure do not show astrogliosis. Previously we have shown that activation of STAT3 and astrogliosis due to MPTP-induced dopaminergic neurotoxicity is restricted to the striatum and non-target regions of the brain remain unaffected [12]. In agreement with the findings for MPTP, brain regions not damaged by METH, KA, and TMT in our mouse models, based on other indices of damage such as Fluoro-Jade staining and argyrophilia ([38]; data not shown), did not show an activation of STAT3 or induction of GFAP (with the exception of a few very minor statistically significant changes) (Fig. 4). These observations provide a further link to the activation of STAT3 as an early biomarker of astrogliosis.

### STAT3 activation, like GFAP up regulation, results from damage associated with multiple models of neurotoxicity: evidence from neuroprotective agents

**Figure 5:**
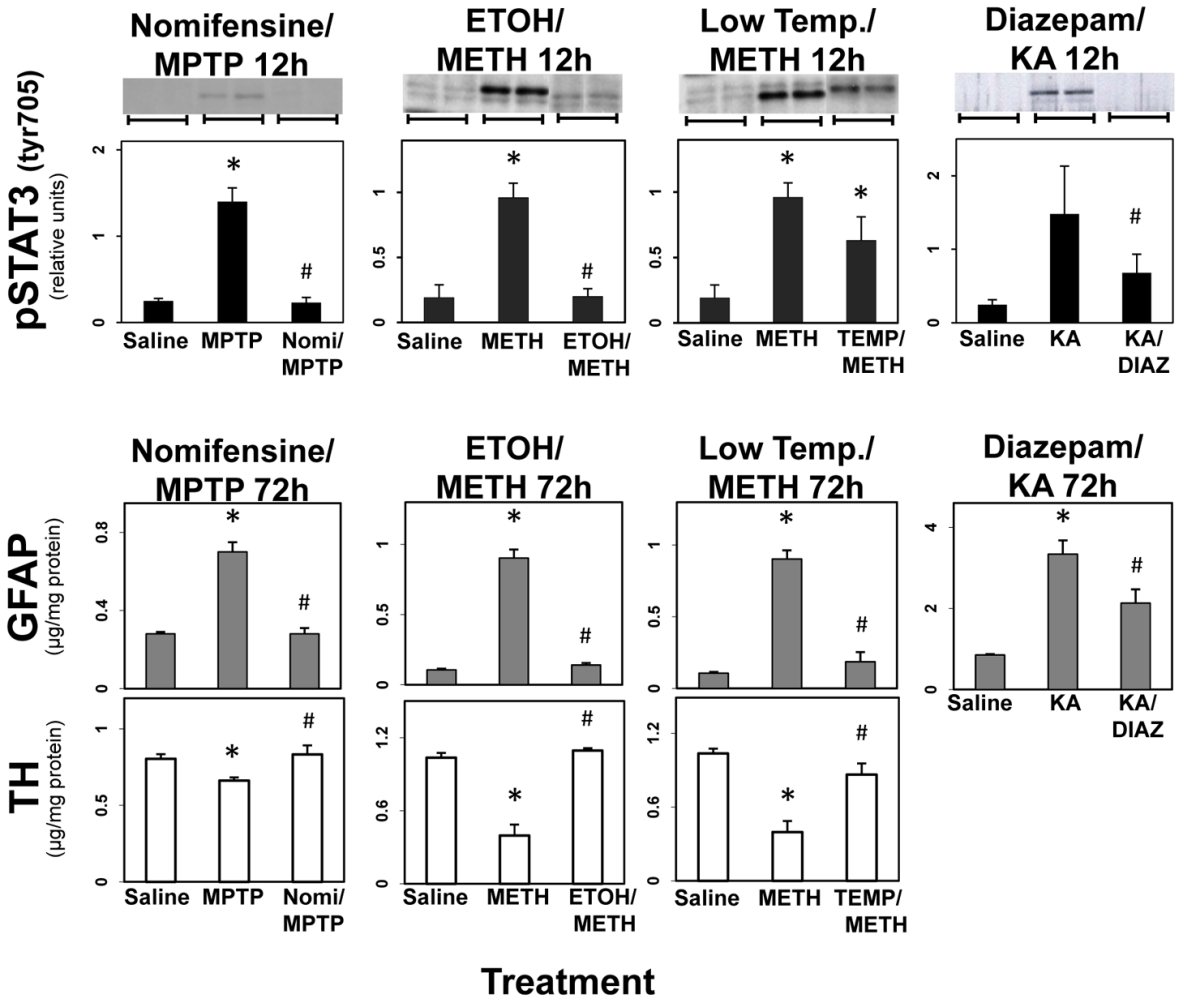
STAT3 activation, like GFAP up regulation, results from damage associated with multiple models of neurotoxicity: evidence from neuroprotective agents. Mice were administered MPTP, METH, or KA alone or after pretreatment with nomifensine (for MPTP), ethanol or low ambient temperature (for METH) or diazepam (for KA). Mice were killed at 12 hours post dosing by focused microwave irradiation for analyses of pSTAT3^tyr 705^ by quantitative immunoblotting or were killed by decapitation at 72 hours post dosing for analyses of GFAP and TH by ELISA. Striatal samples were assayed after MPTP and METH and hippocampal samples were assayed after KA. Representative immunoblot data from two different animals for each treatment condition are presented above the quantitative data obtained from scans. Except for the representative immunoblot duplicates, all data points represent mean ± SEM, N = 5. Statistical significance was measured by two-way ANOVA with Fisher's LSD Method of post hoc analysis. * denotes statistical significance of at least p<0.05 for the neurotoxicant alone or pretreated groups compared to saline controls and # denotes statistical significance of at least p<0.05 for neuroprotectant pretreated groups compared to neurotoxicant only exposed groups. See Methods for dosing regimen (single dose METH).

If activation of STAT3, like astrogliosis, is linked to damage, then neuroprotective agents that prevent damage should prevent the activation of STAT3 and astrogliosis. Preventing MPP^+^ access to striatal dopaminergic nerve terminals by pretreatment with the dopamine transporter antagonist, nomifensine, is known to protect against MPTP-induced dopaminergic neurotoxicity. In agreement with our previous findings [12], pretreatment with nomifensine prevented the activation of STAT3, induction of astrogliosis (as measured by increases in GFAP levels) and the dopaminergic neurotoxicity (decrease in TH) associated with MPTP-induced neurotoxicity (Fig. 5). Lowering core temperature by prior treatment with ethanol or by lowering ambient temperature is known to protect against METH-induced neurotoxicity [36]. These interventions prevented the activation of STAT3, the induction of astrogliosis and dopaminergic neurotoxicity due to METH (Fig. 5). Diazepam can block or attenuate neurotoxicity due to kainate [39]. Pretreatment with diazepam (30 mg/kg, i.p.) blocked kainate-induced activation of STAT3 and markedly attenuated kainate-induced astrogliosis (Fig. 5). Together, these observations indicate that activation of STAT3, like the induction of astrogliosis, results from neuronal damage caused by multiple mechanistically diverse neurotoxicants that damage different regions of the brain.

### Conditional deletion of STAT3 blocks neurotoxicant-induced GFAP up regulation

**Figure 6:**
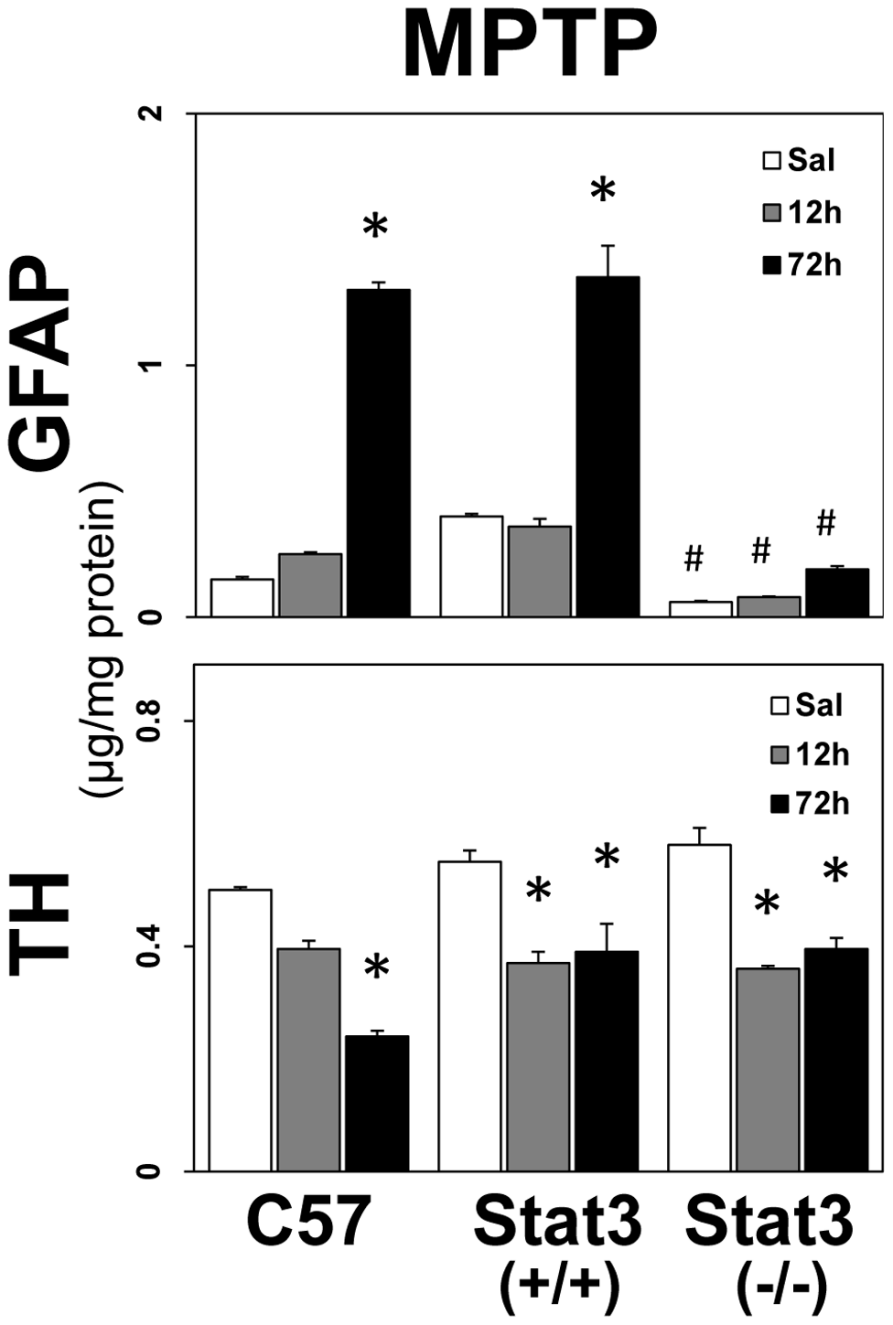
Conditional deletion of STAT3 blocks neurotoxicant-induced GFAP up regulation. Wild type (C57), Stat3 (+/+) or Stat3 (−/−) mice were administered MPTP (12.5 mg/kg, s.c.) and killed at 12 and 72 hours post dosing. Striatal GFAP and TH were measured by ELISA. All data points represent mean ± SEM, N = 5. Statistical significance was measured by two-way ANOVA with Fisher's LSD Method of post hoc analysis. Statistical significance of at least p<0.05 for the MPTP-exposed groups is denoted by * as compared to wild-type controls (C57) and by # as compared to the wild-type (C57) and Stat3+/+ groups.

Data presented in the previous figures provide a correlational implication for a role of STAT3 in the induction of astrogliosis. If STAT3 signaling plays a causal role in neurotoxicant-induced astrogliosis, then its ablation in astrocytes by conditional gene deletion should block the astroglial response to neurotoxic insult. Using the same STAT3 conditional knock-out (CKO) mice previously shown to attenuate the up regulation of GFAP and associated astrogliosis in a spinal cord injury model [17], we found that basal expression of GFAP in striatum was attenuated and the enhanced expression of GFAP in response to MPTP-induced dopaminergic neurotoxicity was markedly attenuated (Fig. 6). The lack of an induction of GFAP in response to MPTP was unlikely to be related to an effect of the conditional STAT3 deletion on MPTP-related dopaminergic neurotoxicity because tyrosine hydroxylase, a marker of dopaminergic nerve terminals in striatum, was decreased to the same degree in CKO mice treated with MPTP.

### STAT3 activation is preceded by enhanced expression of proinflammatory ligands upstream of JAK2/STAT3 in multiple models of neurotoxicity

**Figure 7:**
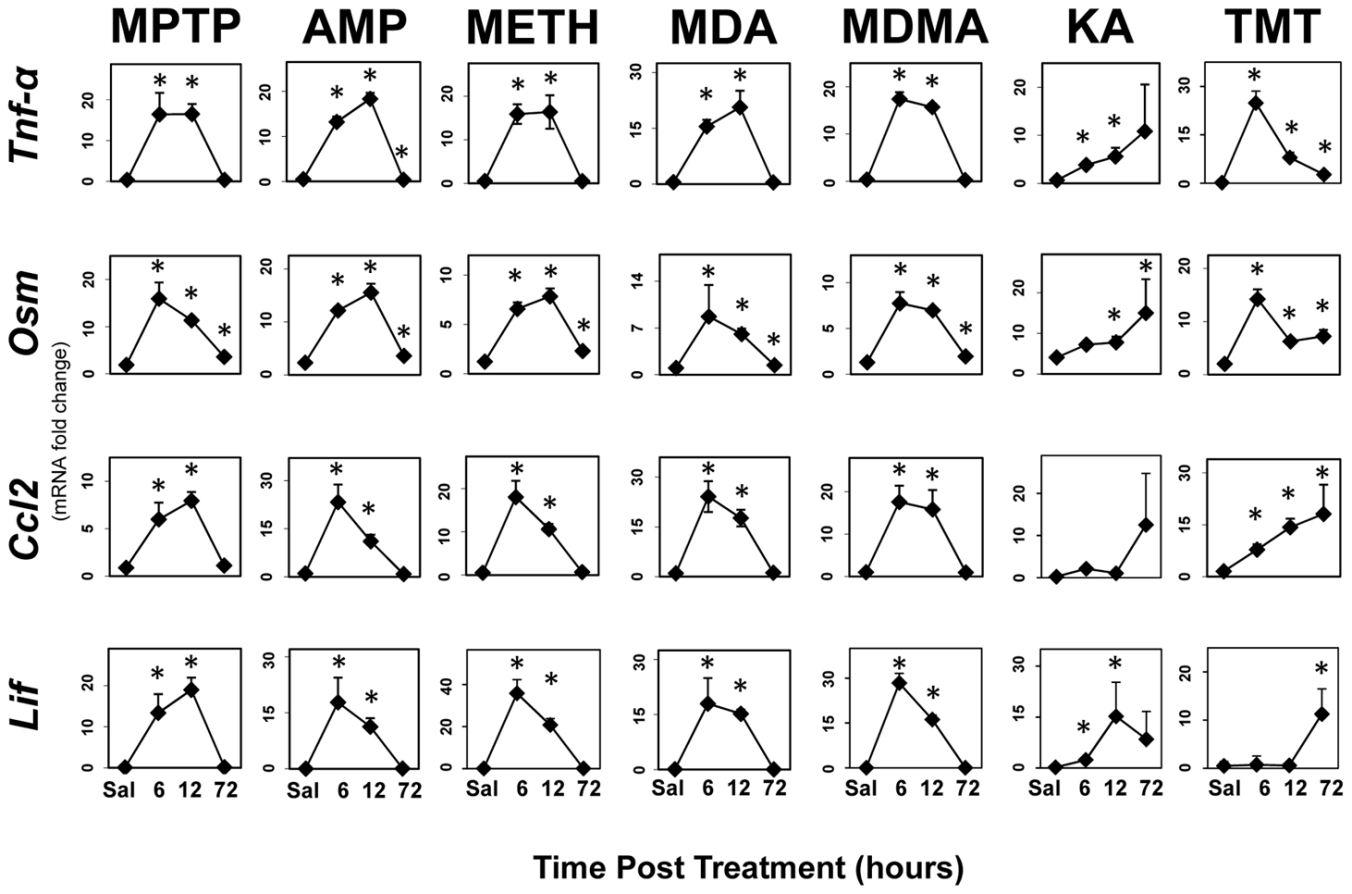
STAT3 activation is preceded by enhanced expression of proinflammatory ligands in multiple models of neurotoxicity. Mice were administered MPTP, AMP, METH, MDA, MDMA, KA and TMT with saline (0.9%) as a control and were killed at 6, 12 and 72 hours post dosing. Total RNA was prepared from striatum (MPTP, AMP, METH, MDA and MDMA) or hippocampus (KA and TMT) for qRT-PCR analysis of *Tnf-α*, *Osm*, *Ccl2* or *Lif*. All data points represent mean ± SEM, N = 5. Statistical significance was measured by one-way ANOVA with Fisher's LSD Method of post hoc analysis. Statistical significance of at least p<0.05 for the neurotoxicant exposed groups in comparison to saline controls is denoted by an asterisk. See Methods for dosing regimen (multi-dose METH).

It is well known that cytokines can activate the JAK2/STAT3 pathway (see reviews: [40], [41]). Up regulation of gp130-and related cytokines associated with the activation of the JAK2/STAT3 pathway precedes activation of STAT3 and astrogliosis in the MPTP model of neurotoxicity [12]. These prior observations were suggestive of a signaling role of proinflammatory cytokines in mediating or modulating the induction of astrogliosis. Here we evaluated proinflammatory cytokine/chemokine expression profiles associated with other models of striatal neurotoxicity and determined if similar responses would be observed with hippocampal neurotoxicity models. Rapid and large-fold up regulation of *Tnf-α*, *Osm*, *Ccl2* and *Lif* were confirmed for MPTP and METH neurotoxicity exposures (Fig. 7). In addition to METH, AMP and its substitutes, MDA and MDMA, also caused the rapid induction of these same proinflammatory signals. The time course of these signaling events preceded the time course for activation of STAT3 and the induction of *Gfap* mRNA and GFAP protein (see Fig. 1). Similarly, the hippocampal neurotoxicants, KA and TMT, also resulted in enhanced expression of mRNA for the same cytokines/chemokines (Fig. 7), prior to the onset of the activation of STAT3 and the induction of *Gfap* mRNA or GFAP protein (Fig. 2). Together, these data indicate that mechanistically diverse neurotoxic exposures damaging two different brain regions all result in the enhanced expression of proinflammatory cytokines upstream of STAT3 prior to induction of GFAP and associated astrogliosis.

### Enhanced expression of proinflammatory ligands results from damage associated with multiple models of neurotoxicity: evidence from neuroprotective agents

**Figure 8:**
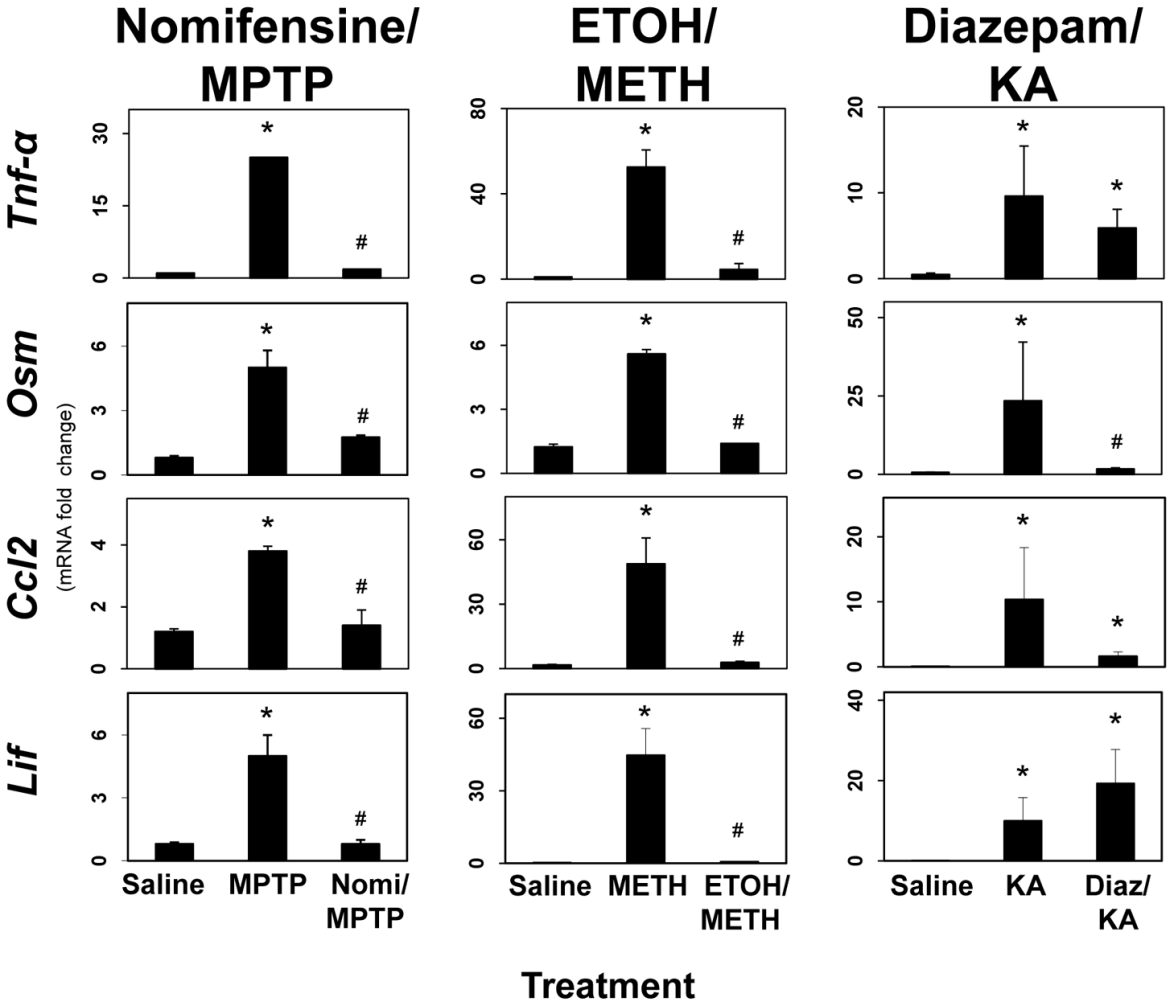
Enhanced expression of proinflammatory ligands results from neurotoxicity-induced damage: evidence from neuroprotective agents. Mice were administered MPTP, METH, or KA alone or after pretreatment with nomifensine (for MPTP), ethanol (for METH) or diazepam (for KA). Mice were killed at 12 hours post dosing. Total RNA was prepared from striatum (MPTP and METH) or hippocampus (KA) for qRT-PCR analysis of *Tnf-α*, *Osm*, *Ccl2* or *Lif*. All data points represent mean ± SEM, N = 5. * denotes statistical significance of at least p<0.05 for the neurotoxicant alone or pretreated groups compared to saline controls and # denotes statistical significance of at least p<0.05 for neuroprotectant pretreated groups compared to neurotoxicant only exposed groups. Statistical significance was measured by two-way ANOVA with Fisher's LSD Method of post hoc analysis. See Methods for dosing regimen (single dose METH). Data for *Osm* and *Lif* in the MPTP groups was taken from Fig. 8 in Sriram et al., (2004) [12].

If enhanced expression of proinflammatory cytokines/chemokines upstream of STAT3 is linked to damage, then neuroprotective agents that prevent damage should prevent enhanced expression of these proinflammatory mediators, as well as activation of STAT3 and astrogliosis. To address this question, we again used nomifensine, ethanol and diazepam to protect against MPTP, METH and KA neurotoxicity, respectively (Fig. 8). Pretreatment with nomifensine and ethanol completely blocked the enhanced expression of *Tnf-α*, *Osm*, *Lif* and *Ccl2* associated with MPTP and METH neurotoxicity, respectively. Diazepam blocked the expression of *Osm* in the KA neurotoxicity model. *Lif* was not affected by diazepam in the KA model. While these data, in general, are consistent with a relationship among the expression of proinflammatory mediators and damage resulting from MPTP, METH and KA, the lack of significant protection by diazepam against KA induced expression of *Tnf-α* and *Ccl2* demonstrate a lack of complete concordance. Moreover, the complete lack of protection of diazepam against expression of *Lif* in the KA model is not consistent with the at least partial protection of diazepam against KA-induced activation of STAT3 and elevation in GFAP (Fig. 5), i.e. the diazepam protection data for KA may indicate that the proinflammatory response can be separated from the damage response. Data for nomifensine neuroprotection against induction of *Osm* and *Lif* in the MPTP model were taken from Sriram et al., (2004) [12].

### Anti-inflammatory treatment with CORT suppresses the enhanced expression of proinflammatory ligands upstream of STAT3 in multiple models of neurotoxicity

**Figure 9:**
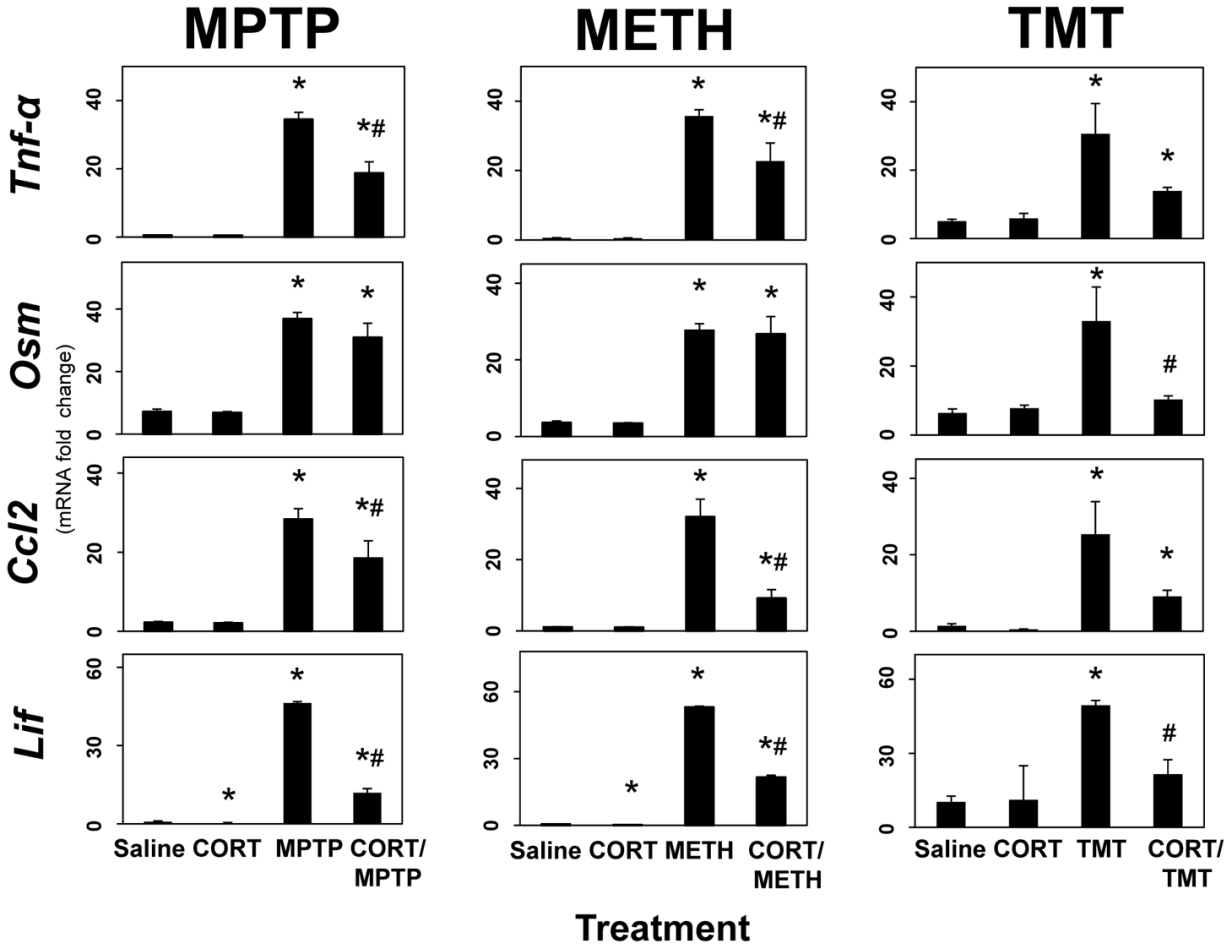
Anti-inflammatory treatment with CORT suppresses enhanced expression of proinflammatory ligands in multiple models of neurotoxicity. Mice were administered MPTP, METH, or TMT alone or after pretreatment with CORT (20 mg/kg, s.c.) 30 minutes earlier. Mice were killed at 12 hours post dosing. Total RNA was prepared from striatum (MPTP and METH) or hippocampus (TMT) for qRT-PCR analysis of *Tnf-α*, *Osm*, *Ccl2* or *Lif*. All data points represent mean ± SEM, N = 5. * denotes statistical significance of at least p<0.05 for the neurotoxicant alone or CORT pretreated groups compared to saline controls and # denotes statistical significance of at least p<0.05 for CORT- pretreated groups compared to neurotoxicant only exposed groups. Statistical significance was measured by two-way ANOVA with Fisher's LSD Method of post hoc analysis. See Methods for dosing regimen (single dose METH).

Because neuroprotective agents that block or suppress neurotoxicity due to MPTP, METH, and KA also block or suppress the enhanced neuroinflammatory effects associated with these agents, it follows that neuroinflammatory mediators upstream of STAT3 play a role in neurotoxicant-induced damage. To begin to examine this issue, we determined if the classic species-specific anti-inflammatory glucocorticoid, CORT, would suppress the neuroinflammatory response associated with diverse neurotoxic exposures (Fig. 9). CORT (20 mg/kg, s.c.) administered 30 minutes prior to MPTP, METH or TMT suppressed the expression of *Tnf-α*, *Osm*, *Ccl2* and *Lif* associated with the neurotoxic effects of these agents. These data suggested that the acute dosage of CORT had the desired anti-inflammatory effect on neurotoxicant-associated neuroinflammation.

### Anti-inflammatory treatment with CORT does not suppress activation of STAT3, GFAP up regulation, or neurotoxicity

**Figure 10:**
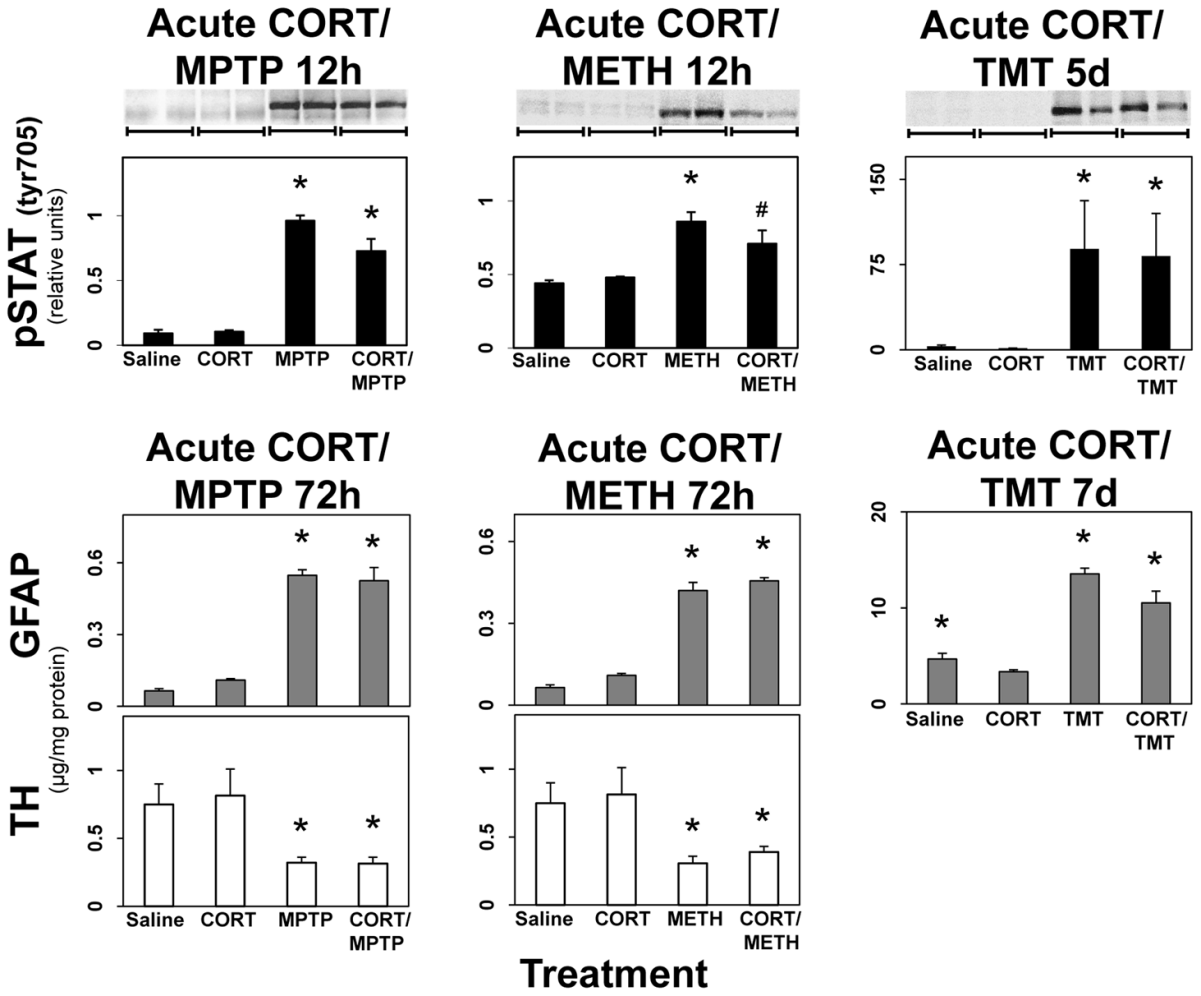
Anti-inflammatory treatment with CORT does not suppress activation of STAT3, GFAP expression, or neurotoxicity. Mice were administered MPTP, METH, or TMT alone or after pretreatment with CORT (20 mg/kg, s.c.) 30 minutes earlier and killed at the post-dosing times indicated. Mice were killed by focused microwave irradiation to preserve steady-state phosphorylation of pSTAT3^tyr 705^ and striatal phosphorylation was analyzed by quantification of scans of pSTAT3^tyr705^ immunoblots. Representative immunoblot data from two different animals for each dosing groups are presented above the quantitative data obtained from scans. Separate groups of mice killed by decapitation were used to prepare total striatal protein homogenates for ELISA of GFAP and TH. All data points represent mean ± SEM, N = 5. * denotes statistical significance of at least p<0.05 for the neurotoxicant alone or CORT pretreated groups compared to saline controls and # denotes statistical significance of at least p<0.05 for CORT- pretreated groups compared to neurotoxicant only exposed groups. Statistical significance was measured by two-way ANOVA with Fisher's LSD Method of post hoc analysis. See Methods for dosing regimens (single dose METH).

To determine if suppression of the neuroinflammatory responses associated with MPTP, METH, and TMT neurotoxicity suppressed activation of STAT3, astrogliosis, and neurotoxicity, we again administered CORT (20 mg/kg, s.c.) prior to MPTP, METH, and TMT (Fig. 10). This anti-inflammatory treatment shown to suppress neuroinflammation in these neurotoxicity models (Fig. 9), failed to affect the activation of STAT3, induction of GFAP, or loss of TH in the MPTP and METH models of striatal neurotoxicity in the mouse, and also failed to suppress the activation of STAT3 and induction of GFAP in the TMT model of hippocampal neurotoxicity in the rat (Fig. 10). Together with the data in Fig. 9, these findings indicate that damage caused by diverse neurotoxicants induces neuroinflammation but that such neuroinflammation is not necessarily responsible for the damage-associated activation of STAT3, astrogliosis or neurotoxicity. The data obtained for MPTP and METH are consistent with our data showing suppression of neuroinflammation by minocycline, a non-traditional immunosuppressant [42], in the MPTP and METH models without affecting neurotoxicity [27].

### Pro-inflammatory treatment with LPS results in enhanced expression of proinflammatory ligands, and a CORT-suppressible activation of STAT3, but not GFAP up regulation

**Figure 11:**
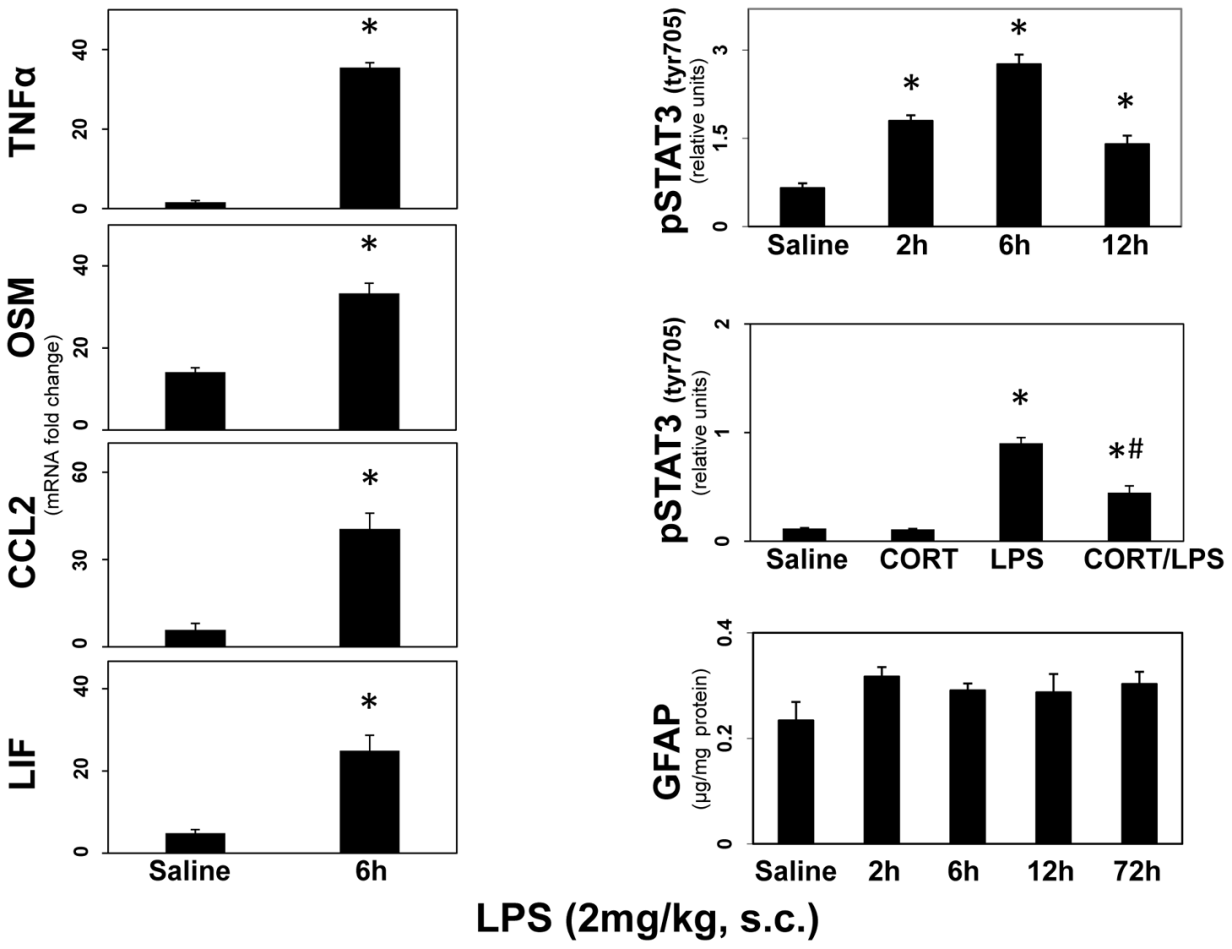
LPS enhances expression of proinflammatory ligands, CORT-suppressible activation of STAT3, but not expression of GFAP. Mice were administered LPS (2 mg/kg, s.c.), CORT (20 mg/kg) or CORT (20 mg/kg) 30 minutes prior to LPS (2 mg/kg). Mice were killed at the post-dosing times indicated or at 6 hours post dosing for the LPS and CORT/LPS groups analyzed for pSTAT3^tyr 705^. Mice were killed by focused microwave irradiation to preserve steady-state phosphorylation of pSTAT3^tyr 705^ and striatal phosphorylation was analyzed by quantification of scans of pSTAT3^tyr705^ immunoblots. Separate groups of mice were used to prepare total striatal RNA from one side of the brain for qRT-PCR analysis of *Tnf-α*, *Osm*, *Ccl2* or *Lif* and to prepare total striatal protein homogenates from the other side of the brain for ELISA of GFAP. All data points represent mean ± SEM, N = 5. * denotes statistical significance of at least p<0.05 for the LPS alone and CORT/LPS groups compared to saline and # denotes statistical significance of at least p<0.05 for CORT- pretreated LPS group compared to LPS only exposed group. Statistical significance was measured by one or two-way ANOVA. Where significant differences were found, Fisher's LSD Method of post hoc analysis was performed. See Methods for other details on dosing regimens.

**Figure 12:**
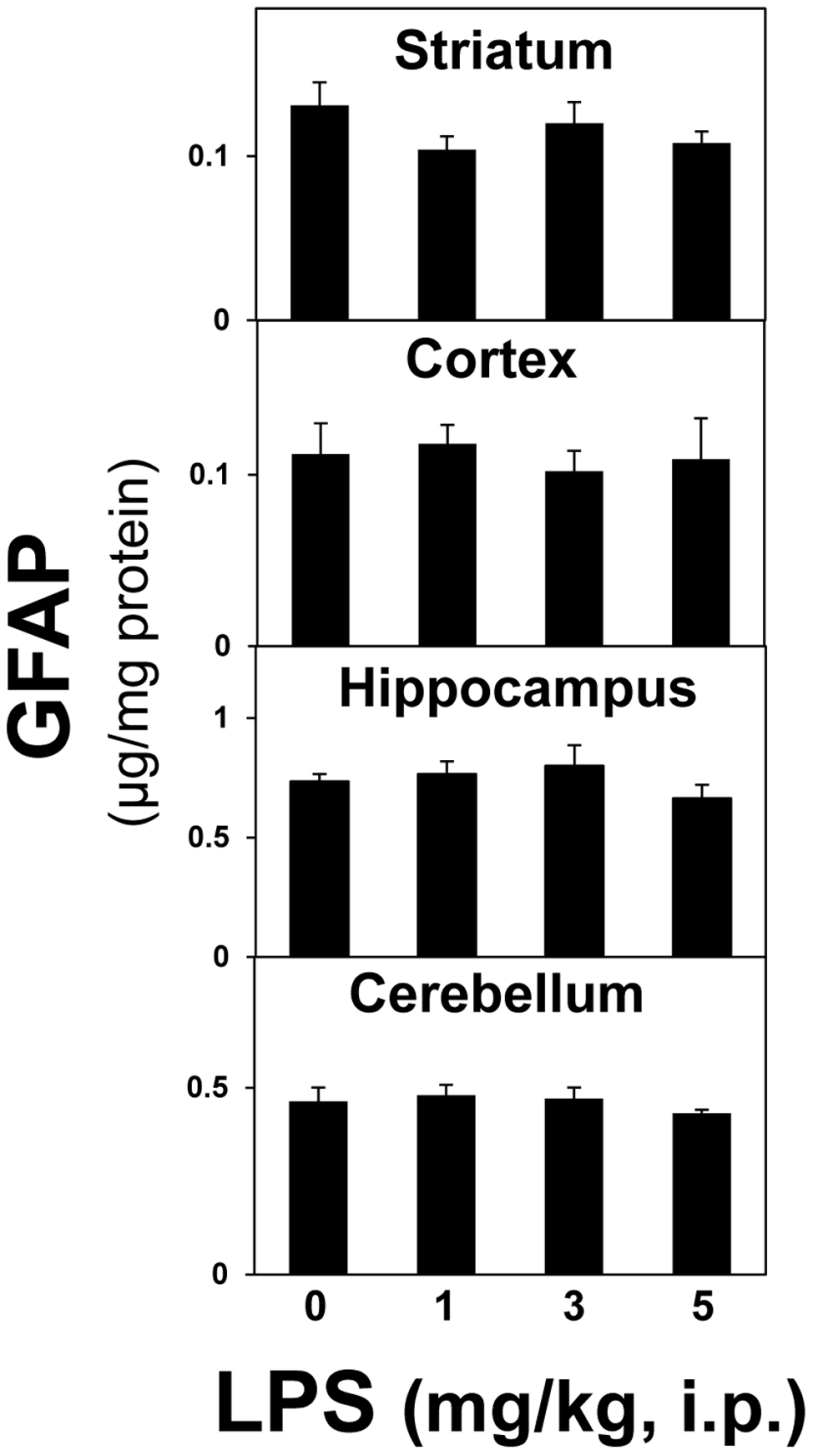
Pro-inflammatory treatment with peripherally injected LPS does not result in increased levels of GFAP at 24 h. Mice were administered LPS (1–5 mg/kg, i.p.) and killed at 24 hours post dosing. Total brain region homogenates were assayed for GFAP by ELISA. All data points represent mean ± SEM, N = 5. Statistical significance was measured by one-way ANOVA. See methods for dosing regimen.

Taken together, the previous data indicate that damage due to exposure to diverse neurotoxicants initiates neuroinflammation but the observed neuroinflammatory responses were not linked to activation of STAT3 and associated astrogliosis. Given these observations, it seemed possible that activation of STAT3 also could be dissociated from damage and astrogliosis, despite the fact that multiple neurotoxicity models result in STAT3 activation temporally linked to induction of GFAP. To examine this possibility, we employed an acute exposure to the known inflammogen, LPS, to induce neuroinflammation (Fig. 11). Our prior experience suggested that acute administration of LPS did not cause neurotoxicity or astrogliosis in any brain region (data not shown). Acute LPS (2 mg/kg, i.p.), as expected, resulted in enhanced expression of the proinflammatory cytokines/chemokines, *Tnf-α*, *Osm*, *Ccl2* and *Lif* (Fig. 11); data are shown for mouse striatum, but similar results were observed for other brain areas (data not shown). LPS also activated STAT3 over a 12-hr post exposure period. The activation of STAT3 by LPS, unlike the activation associated with multiple models of neurotoxicity, was suppressible by acute pretreatment with CORT. The neuroinflammation and activation of STAT3 resulting from acute administration of LPS did not affect the expression of GFAP in any brain region over a 72-hour time period (striatal data shown). It was possible that the dosage and route of administration of LPS we used were not sufficient to affect GFAP, as has been previously reported [5]. Therefore, we administered LPS at 1–5 mg/kg, i.p. and assayed GFAP levels in multiple brain regions at 24 hours post exposure (Fig. 12). GFAP was not affected at any dose in any region examined (Fig. 12). Together, these data serve to indicate that “acute phase” neuroinflammation caused by LPS can activate STAT3 without resulting in the classic feature associated with astrogliosis (increased GFAP), findings consistent with gene expression events shown for LPS, *in vitro*, without accompanying increases in GFAP [43]. Thus, the STAT3 pathway appears to serve as a dual “switch” for mediating acute neuroinflammatory responses separate from its role in mediating damage-induced astrogliosis.

## Discussion

**Figure 13:**
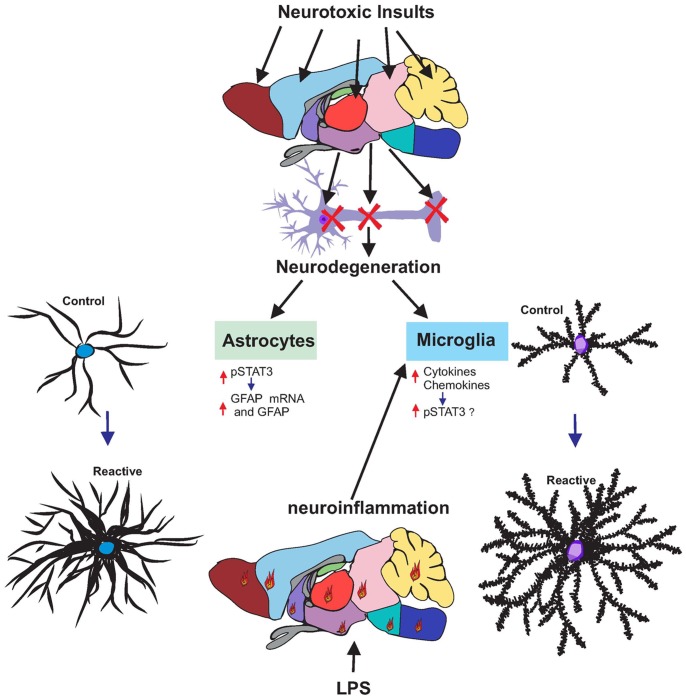
Different neurotoxic insults damage neurons in different brain areas, leading to astrocyte and microglia activation. Activation of STAT3 (pTYR 705) represents a signaling event common to neurotoxic insults and neuroinflammation. Neurotoxicity-related damage results in astrogliosis dependent on activation of STAT3 but does not require upstream signaling from proinflammatory cytokines and chemokines. Suppression of this neuroinflammatory response to neurotoxicity does not affect expression of GFAP or pSTAT3. LPS causes brain-wide neuroinflammation (represented by flames) characterized by activation of microglia and elaboration of proinflammatory cytokines/chemokines but not neurodegeneration. Neuroinflammation-related activation of microglia due to LPS does not lead to astrogliosis but also is associated with activation of STAT3 suppressible by glucocorticoids. Neuroinflammation likely activates a separate STAT3 pathway, perhaps in microglia. Identification of upstream effectors in these STAT3 pathways will aid in defining and manipulating signal transduction events that likely play roles in repair and neuroimmune responses.

We demonstrate that the STAT3 pathway in astrocytes is activated in diverse models of neurotoxicity-initiated astrogliosis, regardless of the underlying mechanism of damage or the regional, cellular or subcellular targets affected by each neurotoxicant. Activation of STAT3 (phosphorylation at Tyr705) ensues rapidly after the onset of neurotoxicity and prior to the induction of GFAP, a STAT3 regulatory target [44], [45]. Activation of STAT3 and subsequent induction of GFAP results from damage to neuronal targets, not effects on astrocytes themselves, as protecting neuronal targets from neurotoxicity also protects against activation of STAT3 and enhanced expression of GFAP. Consistent with these observations, STAT3 activation is restricted to the targeted brain region, associated with translocation to the nucleus of astrocytes after MPTP [12], METH and KA and its deletion in a conditional knock-out abolishes the expression of GFAP in the MPTP neurotoxicity model. Thus, based on our data obtained with the multiple neurotoxicants examined to date, pSTAT3^tyr705^ appears to be a key signaling event underlying astrogliosis resulting from divergent types of neurotoxicity affecting different CNS targets. The “acute phase” response [46] exemplified by systemic exposure to endotoxin (LPS) [47] also results in neuroinflammation and activation of the STAT3 pathway [48] but, in our hands, without the up regulation of GFAP, a hallmark of reactive astrogliosis, findings suggestive of a dissociation of the early neuroinflammatory/microglial response to systemic infection from the astroglial reaction linked to underlying neural damage. These findings are summarized in Figure 13. In aggregate, our findings not only support a general role of pSTAT3^tyr705^ in astrogliosis signal transduction, but also suggest that pSTAT3^tyr705^ serves as an early and broadly applicable biomarker of neurotoxicity. Activation of STAT3 in response to systemic inflammation may subserve a different role distinct from astroglial activation in response to neural damage.

### Relationship among enhanced expression of proinflammatory mediators, activation of STAT3 and astrogliosis

Our prior data with the MPTP neurotoxicity model was suggestive of a role of proinflammatory cytokines/chemokines as upstream effectors in the astrocytic JAK2/STAT3 cascade [12]. STAT3 is a major signaling component in inflammatory responses [16], [21], [49]–[51] and proinflammatory cytokines that signal through gp130 cause its dimerization and activation of JAK2, which in turn phosphorylates Y705 on STAT3, triggering its translocation to the nucleus [52], [53]. Consequently, in the present study with multiple neurotoxicants, as well as with the prior one limited to MPTP, we examined the expression of gp130 ligands known to activate the STAT3 pathway. In agreement with others [54], we indeed found large-fold increases in the gp130 ligands, *Osm* and *Lif*, across all of the diverse neurotoxic exposures with expression time courses preceding those of pSTAT3^tyr705^ and of GFAP, observations suggestive of upstream effector roles for these ligands in the activation of the STAT3 signaling pathway and astrogliosis. Large increases also were seen in *Tnf-α* and *Ccl2* across the various neurotoxicity models and these proinflammatory mediators could influence STAT3 signaling through various cross-talk pathways (e.g., [13], [55]). Our findings indicate that STAT3 is a participant in neurotoxicity-induced astrogliosis because protecting against neurotoxicity in multiple models blocks or greatly attenuates activation of STAT3 in addition to the induction of GFAP. Moreover, eliminating STAT3 in a conditional knockout nearly abolishes neurotoxicity-related expression of GFAP. Roles for gp130 ligands in the induction of astrogliosis are less clear.

The data for all the neurotoxic insults we employed show a consistent up regulation of *Tnf-α*, *Osm*, *Ccl2* and *Lif* with a temporal profile that precedes activation of STAT3 and an enhanced expression of GFAP, findings consistent with microglial activation in response to neurotoxicity [56]. While these effects clearly are related to neurotoxicity because they are blocked or attenuated by neuroprotectants, our data do not define the source of their expression or demonstrate their role in STAT3 signaling, beyond a correlative time course. To address the potential linkage between cytokine/chemokine expression and astrogliosis we suppressed the proinflammatory responses resulting from MPTP and METH neurotoxicity by pretreating with the tetracycline-like anti-inflammatory compound, minocycline [27]. While minocycline was effective in down-regulating the expression of all cytokines/chemokines examined [27], it did not affect MPTP- or METH-induced damage to dopaminergic nerve terminals, nor did it alter astrogliosis as assessed by quantification of GFAP. These data were suggestive of a dissociation of damage-related microglial activation responses from subsequent influences on neurotoxicity or the induction of astrogliosis. The data obtained in the present study support this line of reasoning because suppression of the expression of *Tnf-α*, *Osm*, *Ccl2,* and *Lif* by acute pretreatment with the classic glucocorticoid anti-inflammatory hormone, CORT, did not affect activation of STAT3, or induction of GFAP in MPTP, METH and TMT models of neurotoxicity. Suppressing neuroinflammation with CORT also failed to suppress neurotoxicity due to MPTP and METH, observations consistent with our prior finding with minocycline in these models [27]. These data show that, at least for the case of relatively selective neurotoxicity, damage results in neuroinflammation but this constellation of proinflammatory responses does not lead to activation of the STAT3 pathway or the induction of astrogliosis, nor does neuroinflammation contribute to neurotoxicity. These findings imply that neurodegeneration-induced microglial activation can occur independently of astroglial activation and that neurodegeneration causes neuroinflammation but neuroinflammation does **not** necessarily contribute to neurodegeneration. Indeed, this view is consistent with a role of acute neuroinflammation in the initiation of regenerative responses to neural injury [57], [58].

### Relationship among neuroinflammatory responses to LPS, activation of STAT3 and astrogliosis

Systemic LPS results in an acute phase response that includes activation of CNS microglia [59] and the brain-wide elaboration of proinflammatory cytokines and chemokines (e.g., [60]). Activation of STAT3 in the CNS also has been reported after systemic administration of LPS [48], [61], [62]. Because the relationship among neuroinflammation, STAT3 activation and astrogliosis, in the absence of CNS damage remains unclear, we examined whether LPS-induced neuroinflammation would activate STAT3 and result in up regulation of GFAP as a marker of astrogliosis. Large-fold increases in expression of the four proinflammatory mediators affected by our panel of neurotoxicants also were seen after LPS, but an activation of STAT3 followed without an induction in GFAP. Moreover, unlike the activation of STAT3 and induction of GFAP seen after neurotoxic exposures, suppression of pSTAT3 levels could be achieved with anti-inflammatory pretreatment with CORT. While evidence has been presented suggesting that LPS can over longer times cause neural damage [5], [63]–[66] and accompanying astrogliosis [67]–[69], our findings here and previously [70], are consistent with an acute and subacute elaboration of cytokines and chemokines as a component of an innate immune response in brain [47], [71]. These neuroinflammatory effects of LPS may reflect a direct action on astrocytes without up regulation of GFAP [43] over acute and subacute times of 2 to 72 hours, or alternatively may reflect an effect on STAT3 in microglia (Fig. 13). Microglial activation responses, e.g. those mediating sickness behavior [22] represent neuroimmune signaling that occur in the absence of brain damage [59] and would not, therefore, be expected to be accompanied by astrogliosis. Subsequent changes in GFAP at more chronic times after LPS may reflect indirect reactive responses of astrocytes to long term effects of LPS on other cells, including potential neurodegenerative effects.

In aggregate, our data for neurotoxic exposures add to the mounting evidence from other injury models showing that STAT3 signaling plays a dominant role in reactive gliosis [12], [72]–[74]. While brain damage also initiates neuroinflammation, as noted above, this latter response does not initiate astrogliosis. Nevertheless, CORT suppressible neuroinflammation in the absence of damage may activate STAT3, perhaps in other cell types besides astrocytes (e.g., microglia), a possibility that could be addressed in future studies using the astrocyte specific STAT3-CKO mice treated with LPS. We can conclude at this juncture that when STAT3 activation is observed in the CNS, it may subserve different roles depending on whether underlying neural damage is involved as opposed to neuroinflammation alone.

Astrocyte heterogeneity now is becoming recognized at both the molecular and cellular levels [75]. This heterogeneity also encompasses astrocytic responses to injury across a variety of damage models [8] Following traumatic injury, for example, astrogliosis phenotypes differ with respect to the distance from the site of injury and with respect to an involvement of a proliferative response associated with astrocytic scarring [15]. These observations likely reflect a gradient of diffusible signals from blood emanating from sites of BBB disruption [15], [76]. STAT3 signaling in astrocytes appears to be an obligatory component underlying all these various reactive astrocytic responses to traumatic brain injury. The neurotoxic insults employed in the present study differ substantially from traumatic brain damage in that BBB is preserved, a proliferative response is not observed even with substantial neuronal damage (e.g. TMT, [77]) and the reactive response is short-lived and resets to pre-exposure levels. Despite these differences compared to traumatic injury, STAT3 activation remains a common feature, findings suggestive of common signaling modules. The glucocorticoid suppressible STAT3 pathway associated with neuroinflammation appears to represent an alternate STAT3 pathway from the one involved in injury (traumatic, neurotoxic, or disease). Clearly, an increased understanding of the diverse signaling molecules responsible for STAT3 activation in the CNS will be required to selectively affect its role in astrogliosis and neuroinflammation.
